# Weight loss practices, perceptions, and eating disorder symptoms among Chinese male adolescent combat sports athletes

**DOI:** 10.3389/fnut.2025.1726260

**Published:** 2026-01-22

**Authors:** Fanjie Meng, Zhao Zhang, Kai Xu, Mengde Lvu, Yuming Zhong

**Affiliations:** 1 Department of Martial Arts and Traditional National Sports, Henan Sport University, Zhengzhou, Henan, China; 2 Krirk University, Bangkok, Thailand; 3 School of Athletic Performance, Shanghai University of Sport, Shanghai, China; 4 School of Biomedical Science and Health, Royal Melbourne Institute of Technology University, Melbourne, VIC, Australia

**Keywords:** body mass loss, dehydration, disordered eating, weight cutting, weight management

## Abstract

**Objectives:**

This study investigated weight loss (WL) practices, perceptions, and eating disorder (ED) symptoms among Chinese male adolescent combat sport (CS) athletes.

**Methods:**

A convenience sampling approach was employed. An adapted Rapid WL Questionnaire and Eating Disorder Examination Questionnaire-8 (EDE-Q8) were used, with 525 and 285 valid responses obtained, respectively. Only participants who had engaged in WL were invited to complete the EDE-Q8.

**Results:**

Fifty-seven percent of participants intentionally engaged in WL practices. The mean habitual WL was 7.2% of body mass (BM), and the highest WL was 9.6% of BM. Significant differences were observed in the age at which WL practices began, highest WL%, and habitual WL% across competitive levels (*p* = 0.024, *p* = 0.009, *p* = 0.010) and sports discipline (*p* = 0.003, *p* = 0.009, *p* = 0.010). Habitual WL% also differed by allocated WL duration (*p* = 0.010). Participants predominantly allocated ≥15 days before the weigh-in for WL (67%). Coaches (78%) were most frequently identified as the primary source of WL guidance. The primary reason reported for engaging in WL was to compete against lighter opponents to increase the likelihood of winning (71%). Most participants perceived that WL had no impact on health (49%), is beneficial to performance (41%), and does not lead to unfair competition (80%). No significant differences in sports discipline, competitive level, or WL practices were observed across athletes with different perceptions. Eighteen percent of participants were classified as exhibiting ED symptoms. Athletes with and without ED symptoms did not differ significantly in habitual WL%. Restraint and global ED scores differed significantly among athletes who allocated different habitual durations for WL (*p* = 0.036, *p* = 0.005). Additionally, habitual WL% was positively correlated with eating concern score (*p* = 0.018).

**Conclusion:**

The magnitude of WL among Chinese male adolescent CS athletes was greater than that reported in previous studies. Most athletes engaged in longer-term WL, allocating ≥15 days, primarily through increased exercise and the use of plastic suits. The higher ED scores were associated with higher habitual WL% and longer allocated WL duration.

## 1 Background

Combat sports (CS) matches are structured around weight classes to allow athletes of comparable size and strength to compete under conditions that are considered fair and safe. Athletes who fail to meet the required body mass (BM) may be disqualified or required to compete in a higher weight class, depending on the specific competition rules and the discretion of the organizing body (1). Within this framework, pre-competition weight loss (WL) is widespread (2, 3), as many CS athletes aim to compete against lighter athletes and optimize their athletic performance (4), and most CS athletes believe that WL can provide them with a competitive advantage (4–7). Despite evidence that WL can impair athletes’ health and performance (1, 8–13), and has even been implicated in fatal incidents (14), its prevalence in CS remains persistently high (3).

Studies have documented WL practices across a wide range of CS athletes, varying by nationality (e.g., Brazil, France, and Australia), sport discipline (e.g., judo, mixed martial arts [MMA], and sambo), competitive level (e.g., high school, university, and elite), age (adolescence and adulthood), and sex (male and female) (15–35). A recent systematic review summarized these studies and found that the majority of athletes engaged in WL (reported rates of 66–100%), most commonly through increased training and gradual dieting, and that habitual WL% ranged from 2–5% of BM during the final 1–2 weeks before competition (3). Significant differences in WL magnitude across sports disciplines have also been reported, with MMA, Muay Thai, and sambo athletes generally showing greater habitual WL% and highest WL% than those in other CS sports (e.g., boxing, sanda, and judo) (3). Recent studies from China further revealed greater WL% and longer allocated WL durations compared with those reported from other countries, underscoring cultural and contextual variations in WL practices (4, 7, 36, 37).

Despite the extensive literature that has provided important insights into WL practices among CS athletes, most investigations have focused on adult populations, with limited evidence specifically addressing male adolescent athletes (15–35, 38–46). To date, only one study has specifically investigated WL practices among male adolescent athletes, and it was limited to judo athletes (21). Even when study samples include adolescent athletes, their data are rarely reported or analysed separately by age group—for example, to determine whether WL practices among adolescents differ by competition level or weight class (18, 23, 24, 30, 33, 40). This gap is notable given that adolescence represents a crucial window for growth, maturation, and long-term athletic development (47, 48). WL during this developmental stage has the potential to interfere with bone growth, height progression, metabolic health, and overall physical development (49–51), particularly when combined with heavy training demands and low energy availability. Additionally, prolonged energy restriction may reduce the basal metabolic rate and increase the risk of future obesity, thereby further compromising physical development and overall health (8, 52).

In addition to these concerns, competing in CS may increase the risk of disordered eating (DE) and eating disorder (ED) symptoms, as athletes often engage in unhealthy and extreme weight control strategies (3, 53, 54). To clarify key terminology for this study: ED represents a clinically diagnosed mental health condition defined by persistent, impairing disturbances in eating behaviors and body image concerns, in accordance with diagnostic guidelines (e.g., the DSM-5-TR). DE refers to a spectrum of irregular and unhealthy eating behaviors that do not meet the full diagnostic criteria for ED. ED symptoms are individual behavioral, cognitive, or physiological manifestations (e.g., self-induced vomiting after eating, laxative abuse, and unrealistic beauty standards) that contribute to, but do not alone constitute, a full ED diagnosis. A recent meta-analysis found that the prevalence of DE among CS athletes (30.4–53.6%) was the second highest, second only to that of gymnastics (55). Despite the high prevalence of DE/ED in CS, the existing literature relies heavily on female cohorts (53, 55–57). Consequently, male adolescents remain underrepresented, and the evidence pertaining to them is both limited and potentially lacks generalisability. Furthermore, the impact of WL on DE/ED symptoms of CS athletes appears to be predominantly short term, and more pronounced in males than in females (7, 53). Previous research has shown that WL variables in CS were associated with dietary restraint score—a subscale score of DE—in males seven days post-competition, with no significant correlations found in females across DE subscales (53). Moreover, DE scores in female athletes were shown to improve during the three-week following competition (53). Similarly, a recent survey revealed that the habitual WL practices of female adolescent CS athletes were not associated with ED symptoms during the off-season (7, 53). However, no study has examined the relationship between habitual WL practices and off-season ED symptoms in male adolescent CS athletes. This gap is particularly concerning given that adolescence represents a critical developmental period in which the chronic effects of WL practices—rather than merely acute WL—are most likely to influence the trajectory of ED symptoms and DE behaviors (47, 48).

Given the lack of research focusing on male adolescent CS athletes and the potential sex differences in the effects of WL on ED symptoms, further investigation is needed. Therefore, the present study aimed to investigate the WL practices, perceptions, and ED symptoms among Chinese male adolescent CS athletes, with the goal of providing novel insights into this underexplored population. The main research questions of this study are: (1) What are the characteristics of pre-competition WL practices (e.g., magnitude of WL, allocated WL duration, methods adopted) among Chinese male adolescent CS athletes? (2) What is the prevalence rate of ED symptoms in this population, and how does this with rates reported in existing literature? (3) Is there a significant association between habitual WL practices (e.g., magnitude of WL, allocated WL duration) and off-season ED symptoms among Chinese male adolescent CS athletes?

## 2 Methods

### 2.1 Experimental approach to the problem

This study employed an observational cross-sectional design, using a questionnaire to assess WL practices and perceptions, as well as to collect data on ED symptoms among Chinese male adolescent CS athletes during the off-season. A convenience sampling strategy was adopted to target the relevant population. The study followed the cross-sectional reporting guidelines outlined by the Strengthening the Reporting of Observational Studies in Epidemiology (STROBE) statement (58) to ensure transparency and consistency in observational research practices.

### 2.2 Participants

A total of 525 participants were included in the study. Informed consent was obtained from all participants and their parents or guardians prior to the study, including both online participants and those participating in person. All data collected were fully anonymized. Ethical approval for conducting the study was obtained from the Ethics Committee of the Shanghai University of Sport (number of approval: 102772023RT170).

The recruitment of participants included online and in-person recruitment. For online recruitment, a questionnaire link was sent to CS team managers via social media (WeChat, Tencent, China), with a request that the content be shared with their athletes (37, 59, 60), Paper-based questionnaires were distributed in person at the largest CS training base in China, with team managers assisting in recruitment during daily team meetings. All athletes who consented to participate in the in-person survey were assembled in a conference room to complete the questionnaire (61). If any questions were unclear, researchers were available to provide detailed explanations, but they were instructed to avoid suggesting or influencing any specific responses. Online respondents could contact the researchers through the phone number provided in the informed consent form to seek assistance and obtain the same explanations as those provided to the in-person participants. During this process, the questionnaire platform enabled respondents to exit the questionnaire filling and save their current answers, allowing them to resume completion at any time. To ensure the integrity of the data, all explanations were strictly factual, and researchers were careful to clarify only the meaning of the questions without guiding the participants toward particular answers. Prior to the application of the questionnaire, participants received an oral briefing for the in-person survey, while written information was provided for the online survey as part of the informed consent procedure.

The inclusion criteria were: (i) consent to participate in the study, (ii) participation in at least one official CS competition within the past 12 months, (iii) currently training as a male CS athlete on a sports team, (iv) age 12–17 years, and (v) during their off-season. A formal *a priori* sample size calculation was not performed. This approach was adopted based on a comprehensive literature review, which indicated that most previous cross-sectional investigations on WL practices in adolescent CS athletes also did not conduct pre-study sample size estimation. Instead, the sample size of the present study was determined by referencing the sample sizes of comparable studies (range: 100–300 participants) and recruiting a larger sample to ensure the statistical adequacy for descriptive and correlation analyses (3). A total of 722 participants completed the questionnaire. The lead author (FJM) pre-screened all responses, and 152 and 45 responses were excluded based on criterion (iv) and (v), respectively, resulting in 525 responses included in the analysis. Only participants who had engaged in WL were invited to complete the Eating Disorder Examination Questionnaire-8 (EDE-Q8). Among these, 525 and 285 participants completed the adapted Rapid Weight Loss Questionnaire (RWLQ) and EDE-Q8, respectively.

### 2.3 Questionnaire development and administration

The WJX web platform (WENJUANXING, www.wjx.cn) was used to construct, distribute, and collect all online questionnaire responses (59, 62). Responses from paper questionnaires were manually entered into the WJX platform by the lead author (FJM). For WL practices and perceptions, this study employed the adapted RWLQ by Zhong et al. (7). The questionnaire consisted of 30 questions and featured four sections: (i) general information, (ii) competition experience, (iii) WL history, and (iv) WL perception. The section on WL history and perception was only available for those who had engaged in WL.

For ED symptom assessment, the Chinese version of EDE-Q8 used in this study was adopted directly from Zhong et al.’s study (7), a short version of the EDE–Q for measuring ED psychopathology in the past 4 weeks. Eight items are rated on a seven-point scale ranging from 0 (*no days/not at all*) to 6 (*every day/markedly*), yielding four subscale scores assessing restraint, shape concern, weight concern, and eating concern. The global ED score represents an average of the four subscale scores, with higher scores indicating greater eating pathology. ED status was identified by a global ED score ≥ 1.68, consistent with the EDE-Q norms for patients with clinical eating disorders, and the reporting of one or more pathologic behaviors including self-induced vomiting, use of laxatives, diuretics, binge eating, on ≥2 days in the past 28 days (63). To further verify its applicability to the target population (male adolescent CS athletes) in the present study, empirical evidence of reliability was supplemented: The internal consistency of the EDE-Q8 was evaluated using Cronbach’s alpha coefficient for the present sample of Chinese male adolescent combat sports athletes. The results showed a Cronbach’s alpha of 0.831, indicating good internal consistency of the scale in this specific population. Data collection occurred from 15 November 2024 to 12 January 2025.

### 2.4 Statistical analyses

All analyses were conducted using SPSS version 27.0 (IBM Corp., Armonk, NY). Descriptive statistics were used to summarize all results. Continuous variables are presented as both median (interquartile range, IQR) and mean (± standard deviation, SD), while categorical variables are presented as frequencies (%). The Shapiro–Wilk test was applied to assess normality. All continuous variables violated parametric assumptions. The inferential statistics analysis used in this study is presented in Appendix A1. Statistical significance was set at *p* < 0.05. Bonferroni corrections were used to adjust for inflated Type I error rates due to multiple comparisons.

Participants were categorized by competitive levels (local, provincial, national), sports discipline (boxing, sanda, taekwondo wrestling, judo), whether their coach guided their WL processes (yes/no), attitude toward impact of WL on health (no impact, detrimental, beneficial), attitude toward impact of WL on performance (no impact, detrimental, beneficial), attitude toward impact of WL on fairness (no impact, leads to unfair competition, unsure), and whether athletes were classified as having an ED (yes/no).

In this study, only the general information, highest WL%, habitual WL%, number of WL cycles in the last year, WR% after weigh-ins, WR/WL ratio, perceptions of the impact of WL, WR% after competitions, age began WL, global ED score, and ED symptom were analyzed using inferential statistics, as these variables are most directly related to our primary research questions and exhibit logical relationships. The frequencies of responses indicating ‘always’ and ‘sometimes’ for WL methods were combined to describe the participants’ primary WL methods, as both represent current use. The formulas used to calculate WL experience, WR% after competition, WR/WL ratio, and influence score were consistent with those employed in the study by Zhong et al. (7).

## 3 Results

The general information of the participants is presented in Table 1. Significant differences were only observed in age began training in current sport between participants who engaged in WL and those who never engaged in WL (*p* = 0.002, *r =* 0.138). Overall, 57% of participants (*n* = 301) reported intentionally engaging in WL for competition, and 69% (*n* = 361) indicated that they had not changed their weight category within the past 2 years. Among the 285 participants who completed the EDE-Q8, 18% (*n* = 52) were classified as presenting ED symptoms. The results of the participants’ WL history and ED scores are presented in Table 2.

**Table 1:** General information of Chinese male adolescent combat sport athletes (*n* = 525).

Variables	Data type	All participants (*n* = 525)	Participants who never engaged in (*n* = 224)	Participants who engaged in (*n* = 301)	*p*
Age (years)	median (IQR)	15.0	15.0	15.0	0.042
		(16.0–14.0)	(16.0–14.0)	(16.0–14.0)	
	mean ± SD	15.2 ± 1.4	15.0 ± 1.4	15.3 ± 1.2	
Stature (cm)	median (IQR)	173	173.0	173.0	0.624
		(178.0–168.0)	(180.0–168.0)	(178.0–169.0)	
	mean ± SD	172.5 ± 10.8	172.9 ± 9.1	172.8 ± 6.9	
Current body mass (kg)	median (IQR)	61	62	61	0.359
		(70.0–54.0)	(74.0–54.0)	(68.0–55.0)	
	mean ± SD	62.8 ± 13.8	64.2 ± 16.3	61.9 ± 10.7	
Off-season body mass (kg)	median (IQR)	60	63.8	60	0.331
		(70.0–54.0)	(73.0–52.3)	(68.0–54.0)	
	mean ± SD	62.4 ± 13.9	63.8 ± 16.2	61.5 ± 10.9	
Age began training in current sport (years)	median (IQR)	12	13	12	0.002
		(13.0–11.0)	(14.0–11.0)	(13.0–11.0)	
	mean ± SD	12.2 ± 1.9	12.5 ± 1.8	12.0 ± 1.8	
Age began competing in current sport (years)	median (IQR)	14	14	14	0.179
		(15.0–13.0)	(15.0–13.0)	(15.0–13.0)	
	mean ± SD	14.1 ± 1.6	14.1 ± 1.4	14.2 ± 1.4	
Competitions participated last competitive season (*n*)	median (IQR)	2	1	2	0.084
		(2-1)	(2-1)	(2-1)	
	mean ± SD	1.8 ± 1.1	1.8 ± 1.0	1.9 ± 1.1	
Medals gained during last competitive season (*n*)	median (IQR)	1	1	1	0.462
		(1–0)	(1–0)	(1–0)	
	mean ± SD	1.0 ± 1.0	0.9 ± 0.9	1.0 ± 1.0	

* = differences between groups (*p* < 0.05). IQR, interquartile range. SD, standard deviation.

**Table 2:** Weight loss history (*n* = 301) and global eating disorder score (*n* = 285) of Chinese male adolescent combat sport athletes who reported engaging in weight loss practices.

Variables	Data type	Boxing (*n* = 60)	Sanda (*n* = 107)	Taekwondo (*n* = 44)	Wrestling (*n* = 27)	Judo (*n* = 12)	Kickboxing (*n* = 51)	*p*	Local (*n* = 68)	Provincial (*n* = 94)	National (*n* = 139)	*p*	Total (*n* = 301)
Age began WL (yr)	median (IQR)	14	15^a^	14^b^	15^a,b^	15	15	0.003*	14	14	15^d^	0.024*	14
		(15–13)	(16–14)	(14–13)	(16–14)	(16–14)	(16–14)		(15–13)	(16–14)	(16–14)		(15–14)
	mean ± SD	14.1 ± 1.1	14.8 ± 1.1	13.9 ± 1.1	15.2 ± 0.9	14.9 ± 1.0	14.6 ± 1.6		14.1 ± 1.5	14.6 ± 1.1	14.7 ± 1.1		14.5 ± 1.2
WL experience (yr)	median (IQR)	0	1	1	0	1	1	/	1	0	1	/	1
		(1–0)	(1–0)	(2–0)	(1–0)	(1–0)	(1–0)		(1–0)	(1–0)	(1–0)		(1–0)
	mean ± SD	0.6 ± 1.2	0.7 ± 0.9	1.1 ± 1.1	0.4 ± 0.5	0.9 ± 1.1	1.2 ± 1.1		1.1 ± 1.1	0.5 ± 0.8	0.8 ± 1.1		0.8 ± 1.0
Highest WL (kg)	median (IQR)	6.0	6.0	6.5	5.0	6.0	4.0	/	4.0	5.5	6.0	/	5.0
		(7.0–4.0)	(8.0–5.0)	(8.8–4.0)	(7.0–4.0)	(8.0–5.0)	(6.0–3.0)		(5.8–3.0)	(7.0–4.0)	(8.0–5.0)		(8.0–4.0)
	mean ± SD	6.0 ± 2.8	6.0 ± 2.3	6.6 ± 2.9	5.6 ± 2.0	6.8 ± 2.8	4.7 ± 2.3		4.56 ± 2.2	6.0 ± 2.5	6.5 ± 2.5		5.9 ± 2.5
Highest WL (%)	median (IQR)	8.9	9.7	10.8	8.3	9.8	6.8^a,b,c^	0.009*	7.0	8.8	10.5^d^	0.009*	9.1
		(12.5–6.7)	(12.9–6.9)	(14.9–8.3)	(9.3–6.7)	(11.4–7.9)	(10.2–4.8)		(9.9–5.2)	(11.4–7.0)	(13.5–8.2)		(12.5–6.7)
	mean ± SD	9.8 ± 4.2	9.9 ± 3.7	11.4 ± 4.3	8.8 ± 3.3	9.8 ± 2.6	7.5 ± 3.4		7.8 ± 3.7	9.3 ± 3.6	10.7 ± 4.0		9.6 ± 4.0
Habitual WL (kg)	median (IQR)	4.0	5.0	5.0	4.0	6.0	3	/	3.0	4.0	5.0	/	4.0
		(5.8–2.0)	(6.0–3.0)	(6.0–3.0)	(5.0–3.0)	(6.0–3.3)	(4.0–2.0)		(4.0–2.0)	(6.0–2.0)	(6.0–3.0)		(6.0–2.0)
	mean ± SD	4.1 ± 2.3	4.8 ± 2.3	5.1 ± 2.6	4.1 ± 2.1	5.6 ± 2.8	3.2 ± 1.8		3.1 ± 1.8	4.3 ± 2.4	5.1 ± 2.3		4.4 ± 2.4
Habitual WL (%)	median (IQR)	6.3	7.4	9.4^a^	6.2	8.0	4.4^b,c^	0.010*	4.5	6.3d	8.3^d,e^	0.010*	6.6
		(9.1–4.2)	(11.1–4.6)	(11.3–6.3)	(8.5–4.2)	(10.0–5.0)	(5.8–3.2)		(6.6–3.1)	(9.1–3.6)	(11.1–5.1)		(9.5–4.1)
	mean ± SD	6.7 ± 3.7	7.9 ± 3.9	9.0 ± 4.1	6.4 ± 2.9	8.1 ± 3.4	4.9 ± 2.4		5.4 ± 3.1	6.7 ± 3.5	8.4 ± 3.9		7.2 ± 3.8
Habitual WL during 60–9 days prior to weigh-in (%)	median (IQR)	2.5	3.9	4.8	2.5	3.3	1.7	/	2.0	2.6	4	/	2.8
		(4.1–1.3)	(6.3–2.1)	(7.4–2.1)	(4.4–0.6)	(4.8–1.2)	(2.9–1.2)		(3.3–1.2)	(4.7–1.2)	(6.3–2.1)		(5.0–1.5)
	mean ± SD	2.9 ± 2.2	4.4 ± 2.8	4.9 ± 3.1	2.6 ± 2.3	3.2 ± 2.1	2.2 ± 1.6		2.6 ± 2.4	3.1 ± 2.3	4.4 ± 2.8		3.6 ± 2.6
Habitual WL during 8–2 days prior to weigh-in (%)	median (IQR)	2.1	2.3	2.1	2.5	2.7	1.3	/	1.3	2.2	2.4	/	2.1
		(3.6–1.0)	(3.8–1.1)	(3.6–1.4)	(3.4–1.9)	(3.4–1.3)	(2.3–0.9)		(2.4–0.9)	(3.5–1.1)	(3.8–1.4)		(3.4–1.1)
	mean ± SD	2.5 ± 2.3	2.6 ± 1.9	2.7 ± 1.9	2.9 ± 2.0	3.1 ± 2.6	1.8 ± 1.3		1.8 ± 1.6	2.5 ± 1.8	2.8 ± 2.1		2.5 ± 2.0
Habitual WL during 1 days prior to weigh-in (%)	median (IQR)	0.9	0.6	0.1	0.9	0.1	0.7	/	0.7	0.9	0.8	/	0.8
		(2.0–0.4)	(1.3–0.1)	(2.1–0.6)	(1.5–0.0)	(1.8–0.5)	(1.2–0.3)		(1.3–0.3)	(1.7–0.4)	(1.6–0.2)		(1.6–0.3)
	mean ± SD	1.2 ± 1.0	1.0 ± 1.1	1.5 ± 1.3	0.9 ± 0.9	1.8 ± 2.1	0.9 ± 0.7		0.9 ± 0.7	1.1 ± 1.0	1.2 ± 1.3		1.1 ± 1.1
WR before competition (kg)	median (IQR)	1.0	1.0	2.0	2.0	3.0	2.0	/	1.0	1.5	2.0	/	1.5
		(2.0–1.0)	(2.0–1.0)	(2.0–1.0)	(2.0–1.0)	(4.8–1.0)	(3.0–1.0)		(2.0–1.0)	(2.0–1.0)	(3.0–1.0)		(2.0–1.0)
	mean ± SD	1.6 ± 1.6	1.8 ± 1.5	2.8 ± 1.5	2.1 ± 1.5	3.0 ± 2.2	2.0 ± 1.5		1.6 ± 1.4	1.9 ± 1.6	2.1 ± 1.7		1.9 ± 1.6
WR before competition (%)	median (IQR)	2.0	2.3	3.6	2.9	5.2	2.9	/	2.2	2.4	3	/	2.6
		(3.3–1.5)	(3.8–1.6)	(4.4–2.2)	(4.6–1.8)	(7.0–1.5)	(4.3–1.5)		(3.7–1.3)	(3.8–1.7)	(4.3–1.8)		(4.1–1.6)
	mean ± SD	2.8 ± 2.7	3.2 ± 2.8	4.1 ± 3.1	3.7 ± 2.8	4.8 ± 3.1	3.4 ± 2.7		2.8 ± 2.4	3.2 ± 2.5	3.8 ± 3.1		3.4 ± 2.8
WL/WR before competition (%)	median (IQR)	33.3	28.6	33.3	50.0	50.0	66.7	0.081	50	38.8	33.3	1.000	40.0
		(66.7–17.5)	(66.7–18.8)	(57.5–20.6)	(75.0–25.0)	(91.7–25.0)	(100.0–37.5)		(93.8–21.3)	(68.8–20.0)	(66.7–20.0)		(66.7–20.0)
	mean ± SD	49.1 ± 52.6	47.9 ± 45.4	51.6 ± 47.7	64.5 ± 54.7	54.2 ± 34.7	70.2 ± 47.3		58.0 ± 43.9	56.6 ± 57.3	50.7 ± 44.0		55.2 ± 56.0
WR after competition (kg)	median (IQR)	3.0	3.0	4.0	4.0	3.0	2.0	/	2.0	3.0	4.0	/	3.0
		(4.0–2.0)	(5.0–2.0)	(5.0–2.0)	(5.0–3.0)	(4.8–2.3)	(3.0–2.0)		(3.0–1.6)	(5.0–2.0)	(5.0–3.0)		(5.0–2.0)
	mean ± SD	3.5 ± 2.4	3.6 ± 1.9	3.9 ± 2.2	4.1 ± 1.8	3.8 ± 2.4	2.7 ± 1.5		2.5 ± 1.3	3.7 ± 2.2	3.9 ± 2.1		3.5 ± 2.0
WR after competition (%)	median (IQR)	4.6	5.6	7.5	6.9	5.1	4.0	/	4	5.1	6.1	/	5.3
		(7.7–3.2)	(8.0–3.8)	(10.1–3.9)	(8.0–4.8)	(7.7–3.8)	(5.9–2.8)		(5.9–2.6)	(7.9–3.4)	(8.8–4.3)		(7.8–3.5)
	mean ± SD	6.0 ± 4.0	6.5 ± 4.0	7.3 ± 4.3	6.5 ± 2.9	5.6 ± 2.6	4.4 ± 2.3		4.3 ± 2.3	6.1 ± 3.5	7.0 ± 4.1		6.1 ± 3.7
Number of WL in the last year	median (IQR)	2	1^a^	2^b^	2	2	1^c^	0.005*	1	2	1	0.144	1
		(3-1)	(2-1)	(3-2)	(2-1)	(3-1)	(2-1)		(2-1)	(2-1)	(3-1)		(2-1)
	mean ± SD	2.0 ± 1.1	1.5 ± 1.4	2.3 ± 1.0	1.9 ± 1.4	2.1 ± 1.1	1.7 ± 1.1		1.5 ± 1.0	1.8 ± 1.0	1.9 ± 1.5		1.8 ± 1.2

Variables	Data type	Boxing (*n* = 60)	Sanda (*n* = 107)	Taekwondo (*n* = 44)	Wrestling (*n* = 27)	Judo (*n* = 12)	Kickboxing (*n* = 51)	*p*	Local (*n* = 68)	Provincial (*n* = 88)	National (*n* = 129)	*p*	Total (*n* = 285)
Restraint score	median (IQR)	0.5	0.5	0.5	0.0	0.5	0.0	1.000	0.0	1.0	0.5	0.234	0.5
		(2.0–0.0)	(2.0–0.0)	(2.8–0.0)	(2.0–0.0)	(2.5–0.0)	(1.0–0.0)		(1.4–0.0)	(2.0–0.0)	(2.5–0.0)		(2.0–0.0)
	mean ± SD	1.4 ± 2.0	1.3 ± 1.6	1.7 ± 2.1	1.2 ± 1.8	1.4 ± 1.7	0.7 ± 1.1		0.8 ± 1.2	1.5 ± 2.0	1.3 ± 1.7		1.2 ± 1.7
Eating concern score	median (IQR)	0.5	0.5	0.0	0.5	0.5	0.3	1.000	0.3	1.0	0.9	1.000	0.5
		(1.4–0.0)	(1.0–0.0)	(1.5–0.0)	(1.5–0.0)	(0.5–0.0)	(0.5–0.0)		(0.5–0.0)	(1.4–0.0)	(1.0–0.0)		(1.0–0.0)
	mean ± SD	1.0 ± 1.5	0.9 ± 1.3	0.8 ± 1.1	1.1 ± 1.5	1.0 ± 1.9	0.6 ± 0.9		0.7 ± 1.3	1.0 ± 1.4	0.9 ± 1.3		0.9 ± 1.3
Shape concern score	median (IQR)	0.0	0.0	0.0	1.0	0.0	0.3	1.000	0.3	0.5	0.0	0.324	0.0
		(1.9–0.0)	(1.1–0.0)	(1.0–0.0)	(2.5–0.0)	(1.0–0.0)	(1.0–0.0)		(1.0–0.0)	(2.0–0.0)	(1.0–0.0)		(1.0–0.0)
	mean ± SD	0.9 ± 1.5	0.8 ± 1.3	0.7 ± 1.1	1.2 ± 1.5	0.6 ± 1.3	0.7 ± 1.0		0.7 ± 1.1	1.1 ± 1.4	0.7 ± 1.3		0.9 ± 1.3
Weight concern score	median (IQR)	0.0	0.5	0.5	1.0	0.0	0.3	0.774	0.5	0.5	0.5	1.000	0.5
		(1.0–0.0)	(1.5–0.0)	(1.5–0.0)	(2.0–0.0)	(1.0–0.0)	(1.0–0.0)		(1.0–0.0)	(1.9–0.0)	(1.0–0.0)		(1.5–0.0)
	mean ± SD	0.7 ± 1.1	1.0 ± 1.3	0.9 ± 1.3	1.1 ± 1.2	0.6 ± 1.2	0.6 ± 0.7		0.7 ± 0.9	1.0 ± 1.3	0.9 ± 1.3		0.9 ± 1.2
Global ED score	median (IQR)	0.5	0.8	0.4	1	0.4	0.4	1.000	0.4	0.8	0.5	0.708	0.5
		(1.6–0.0)	(1.4–1.3)	(1.5–1.3)	(2.0–0.0)	(1.3–0.0)	(0.9–0.0)		(1.0–0.0)	(1.9–0.3)	(1.4–0.0)		(1.5–0.0)
	mean ± SD	1.0 ± 1.3	1.0 ± 1.1	1.0 ± 1.1	1.1 ± 1.1	0.9 ± 1.3	0.6 ± 0.7		0.7 ± 0.9	1.1 ± 1.2	0.9 ± 1.1		0.9 ± 1.1

*Significant differences between groups (*p* < 0.05). ^a^significant different compared to boxing; ^b^significant different compared to sanda; ^c^significant different compared to taekwondo; ^d^significant different compared to local-level; ^e^significant different compared to provincial-level. / = not available. WL, weight loss. WR, weight regain. IQR, interquartile range. SD, standard deviation. ED, eating disorder.

Kruskal–Wallis tests revealed significant differences in the age at which WL practices began (*p* = 0.024, ε^2^ = 0.025), highest WL% (*p* = 0.009, ε^2^ = 0.092), and habitual WL% (*p* = 0.010, ε^2^ = 0.107) across competitive levels; the age at which WL practices began (*p* = 0.003, ε^2^ = 0.110), highest WL% (*p* = 0.009, ε^2^ = 0.076), habitual WL% (*p* = 0.010, ε^2^ = 0.104), and number of WL cycles in the past year (*p* = 0.005, ε^2^ = 0.105) across sport discipline; the habitual WL% (*p* = 0.010, ε^2^ = 0.092) across the allocated WL duration; and restraint score (*p* = 0.036, ε^2^ = 0.043) and global ED score (*p* = 0.005, ε^2^ = 0.061) across allocated WL duration.

Post-hoc analyses of Kruskal–Wallis tests revealed that age at which WL practices began was significantly later among national-level athletes compared with local-level athletes (*p* = 0.007, *r =* 0.211). Wrestlers began WL significantly later than taekwondo (*p* < 0.001, *r =* 0.492) and boxing athletes (*p* = 0.001, *r =* 0.443), and sanda athletes also began WL significantly later than taekwondo (*p* < 0.001, *r =* 0.340) and boxing athletes (*p* = 0.001, *r =* 0.309). The highest WL% was significantly greater among national-level athletes than among local-level athletes (*p* < 0.001, *r =* 0.373). Kickboxers exhibited significantly lower highest WL% than boxing (*p* = 0.030, *r =* 0.307), sanda (*p* = 0.001, *r =* 0.321), and taekwondo athletes (*p* < 0.001, *r =* 0.496). For habitual WL%, national-level athletes showed significantly higher values than provincial (p = 0.001, *r =* 0.210) and local-level athletes (*p* < 0.001, *r =* 0.395), and provincial-level athletes had higher values than local-level athletes (*p* = 0.009, *r =* 0.204). Habitual WL% was significantly lower in kickboxers compared with sanda (*p* < 0.001, *r =* 0.381) and taekwondo athletes (*p* < 0.001, *r =* 0.540), and boxers showed significantly lower values than taekwondo athletes (*p* = 0.044, *r =* 0.292). Athletes who allocated 15–21 days exhibited significantly higher habitual WL% than those who allocated 4–5 days (*p* = 0.016, *r =* 0.292), while those allocating 21 + days showed significantly higher values than athletes allocating 4–5 days (*p* < 0.001, *r =* 0.487), 8–10 days (*p* = 0.028, *r =* 0.305), or 11–14 days (*p* = 0.026, *r =* 0.295). Regarding the number of WL in the past year, taekwondo athletes reported significantly more attempts than sanda (p < 0.001, *r =* 0.435) and kickboxing athletes (*p* = 0.024, *r =* 0.323), while boxers also reported significantly more attempts than sanda athletes (*p* = 0.003, *r =* 0.291). No significant differences were observed in restraint score across the different time allocations for WL. However, the global ED score was significantly higher among athletes allocating 15–21 days for WL compared with those allocating 4–5 days (*p* = 0.048, *r =* 0.264) and 8–10 days (*p* = 0.040, *r =* 0.254).

**Figure 1:**
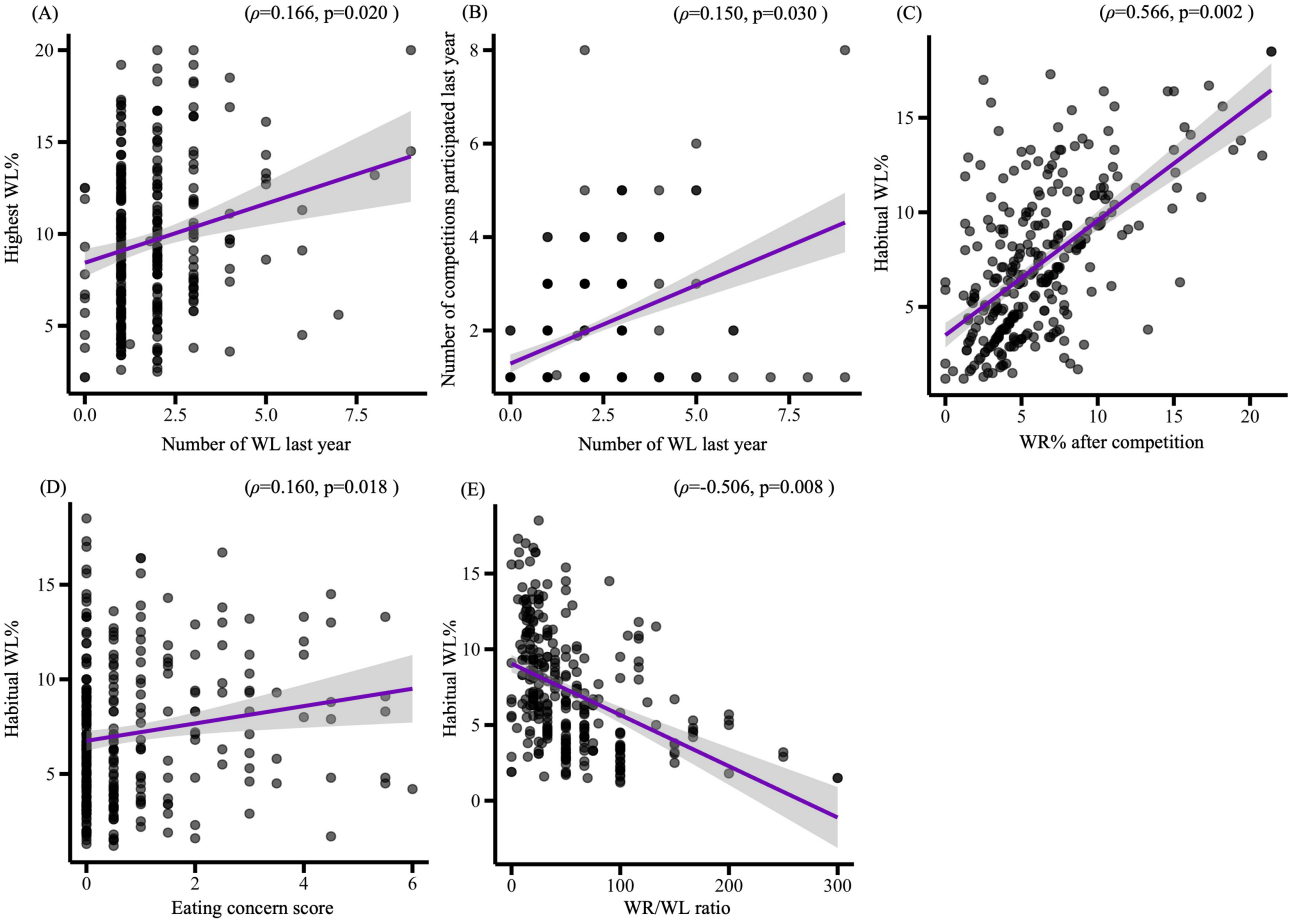
Spearman’s rank correlations between weight loss-related variables and eating disorder variables in male adolescent combat sport athletes. WL, weight loss; WR, weight regain; **(A)** Number of WL cycles in the past year vs. highest WL%. **(B)** Number of WL cycles in the past year vs. number of competitions participated last year. **(C)** Habitual WL% vs. WR% after competition. **(D)** Habitual WL% vs. eating concern, **(E)** Habitual WL% vs. WR/WL ratio.

Spearman’s rank correlation analyses further demonstrated significant relationships. Specifically: the number of WL cycles last year was positively correlated with highest WL% (*ρ* = 0.166, *p* = 0.020) (see Figure 1A) and the number of competitions participated last year (*ρ* = 0.150, *p* = 0.030) (see Figure 1B). Habitual WL% was positively correlated with WR% after competition (*ρ* = 0.566, *p* = 0.002) (see Figure 1C) and eating concern score (*ρ* = 0.160, *p* = 0.018) (see Figure 1D), but negatively correlated with the WR/WL ratio (*ρ* = −0.506, *p* = 0.008) (see Figure 1E).

Mann–Whitney U test revealed no statistically significant differences in the age at which WL practices began (*p* = 0.846, *r =* 0.062), highest WL% (*p* = 0.680, *r =* 0.105), habitual WL% (*p* = 1.000, *r =* 0.075), the number of WL cycles in the past year (p = 1.000, *r =* 0.039), or WR/WL ratio (*p* = 0.722, *r =* 0.021) between groups based on whether coaches guided the WL process. No significant differences were detected in WL experience, habitual WL%, or habitual WL% during the 1-day prior to weigh-in in relation to the presence of ED symptoms (*p* > 0.05).

Chi-square test revealed no significant differences in the perception of the impact of WL on health, performance, or fairness across different sports disciplines or competitive levels (*p* > 0.05).

The majority of participants reported allocating 15–21 days before the weigh-in for WL (40%), followed by 21 + days (27%), 11–14 days (13%), 8–10 days (10%), 6–7 days (5%), 4–5 days (4%), and 1–3 days (1%). Coaches (78%) were most frequently identified as the primary guides for WL, followed by self-guidance (46%), strength and conditioning coaches (9%), doctors, (3%), parents (2%), nutritionists (1%), and other sources (1%). The primary reason reported for engaging in WL was to compete against lighter opponents to increase the chances of winning (71%), followed by optimizing athletic performance (55%), being above usual weight before a competition (39%), “because everyone else is cutting weight, so I have to do it too” (3%), “my coach told me to lose weight, so I have to do it” (2%), and others (1%). Most participants perceived that WL had no impact on health (49%), whereas 30% perceived it as detrimental and 21% as beneficial. Similarly, a majority perceived WL as beneficial to performance (41%), followed by 34% who reported no impact and 25% who considered it detrimental. Regarding fairness, most participants indicated that WL did not lead to unfair competition (80%), whereas 17% were uncertain and 3% perceived it as unfair. The WL methods used by athletes are shown in Table 3, and the influence of different sources on WL practices is shown in Table 4.

**Table 3:** Frequency analysis (%) of the weight loss methods used by Chinese male adolescent combat sport athletes (*n* = 301).

Methods	Always		Sometimes		Almost never		Never used		Do not use any more	
	*n*	%	*n*	%	*n*	%	*n*	%	*n*	%
Gradual dieting	63	21	89	30	55	18	79	26	15	5
Skipping meals	50	17	113	37	46	15	75	25	17	6
Fasting	13	4	22	7	59	20	189	63	18	6
Restricting fluid ingestion	57	19	76	25	55	18	104	35	9	3
Increased exercise	147	49	96	32	25	8	26	9	7	2
Training in a heated room	64	21	54	18	70	23	100	33	13	4
Sauna	14	5	37	12	61	20	175	58	14	5
Training in plastic suits	106	35	94	31	29	10	62	21	10	3
Use plastic suit all-day	21	7	22	7	64	21	179	60	15	5
Spitting	22	7	48	16	40	13	172	57	19	6
Laxatives	4	1	10	3	18	6	254	84	15	5
Diuretics	2	1	4	1	17	6	262	87	16	5
Diet pills	2	1	7	2	15	5	262	87	15	5
Vomiting	14	5	39	13	37	12	193	64	18	6
Hot water immersion	10	3	28	9	29	10	219	73	15	5
Hot saltwater immersion	3	1	12	4	28	9	242	80	16	5
Others	21	7	4	1	15	5	236	78	25	8

**Table 4:** Frequency analysis (%) of the sources of influence on the weight loss practices of Chinese male adolescent combat sport athletes (*n* = 301).

Sources	Very influential		Some influence		Unsure		Little influence		Not influential		IS
	*n*	%	*n*	%	*n*	%	*n*	%	*n*	%	
Other athletes (different sports)	5	2	25	8	20	7	37	12	214	71	158
Other athletes (same sport)	23	8	82	27	19	6	52	17	125	42	242
Doctors	9	3	28	9	34	11	36	12	194	65	173
SC coaches /physical trainer	37	12	59	20	32	11	32	11	141	47	242
Coaches	97	32	82	27	16	5	29	10	77	26	329
Parents	45	15	65	22	28	9	39	13	124	41	257
Nutritionists	14	5	15	5	39	13	26	9	207	69	171
Journal articles	6	2	16	5	32	11	28	9	219	73	154
Book/magazines	7	2	21	7	29	10	30	10	214	71	159
Internet sources	6	2	44	15	29	10	29	10	193	64	184
Others	11	4	6	2	25	8	10	3	249	83	141

SC, strength and conditioning; IS, influence score.

## 4 Discussion

This study investigated the WL practices, perceptions, and ED symptoms of Chinese male adolescent CS athletes. The primary findings include: (i) the prevalence of WL among male adolescent CS athletes is relatively moderate, but the magnitude of WL is high; (ii) no significant differences in sport discipline, competitive level, or WL practices were observed across athletes with different perceptions of WL regarding health, performance, and fairness; (iii) most of the WL practice variables showing significant differences were observed across sport disciplines and competitive levels; and (iv) during the off-season, habitual WL% and allocated WL duration were significantly associated with ED symptoms and scores.

In this study, 57% of participants reported intentionally engaging in WL practices, which is lower than previous findings in CS athletes (3), but broadly comparable to rates observed in studies involving adolescent samples (60–73%) (4, 7, 18, 23, 24, 30, 33, 36, 37, 40), and one study of regional-level adult athletes (59%) (46). The prevalence observed here was also lower than that previously reported among adult CS athletes from China (4, 7, 36, 37). This pattern aligns with earlier findings showing that WL prevalence among adolescent CS athletes is generally lower than that of adults (37). A plausible explanation is that some younger athletes may not yet have begun engaging in WL practices, or that the smaller weight-category increments in adolescent divisions reduce the incentive to engage in extreme WL.

The mean habitual WL of participants was 7.2% of BM (Table 2), higher than that reported in most previous studies of CS athletes (1.8–6.7%) (3, 4, 37), and similar to studies in sambo (7.4%) (27), mixed sport disciplines (7.8%) (7), and lower than studies in Muay Thai (10.6%) (16), sanda (10.8%) (45), and MMA (8.9–13.0%) (17, 25, 31, 44). Similarly, the highest WL was 9.6% of BM, comparable to or higher than most previous studies (4.7–9.6%) (3, 4, 37), and lower than to some of those observed in MMA (10.2–17.5%) (17, 25, 31, 44), sambo (10.6%) (29), Muay Thai (13.9%) (16), visually impaired judo (10.0%) (33), mixed sport discipline (10.3%) (7), and sanda (10.3–10.8%) (36, 45). These results suggest that the habitual and highest WL% of male adolescent CS athletes in the present study were exceptionally high, even higher than the WL% reported in most adult sample studies. Although most athletes allocated more than 2 weeks before the weigh-in for WL, a proportion considerably higher than that reported in other studies (13–31%) (30, 32, 46, 64), prolonged periods of energy deficiency, particularly among 12- to 17-year-old athletes, may still pose serious health risks, such as impaired bone mineralization and stunted height development (8, 52). It is worth noting that heightened competitiveness of national events (where athletes experience greater pressure to gain a competitive edge by reducing a significant amount of BM in national competitions) and more extensive WL experience among national-level athletes likely contribute to the higher WL% observed in this group.

On average, athletes regained 3.4% of BM (corresponding to 55.2% of the reduced BM) between weigh-ins and competitions. Most athletes did not fully regain their BM to habitual levels, likely due to repeated weigh-ins across competition days. Habitual WL% was negatively correlated with the WR/WL ratio, suggesting that athletes with lower WL% tend to regain their BM closer to habitual levels, and smaller WL magnitude is accompanied by sufficient time for BM recovery between consecutive competitions. On average, athletes regained 6.1% of BM within 1 week after competition, with a significant positive correlation observed between habitual WL% and WR% after the competition. These findings suggest that athletes generally attempt to regain their BM to habitual levels within the first week after competition. However, this cyclical weight fluctuation not only reinforces unhealthy weight fluctuations but may also impair growth, endocrine balance, and bone development (54). Therefore, practitioners should closely monitor the athletes’ post-competition diet and regulate weight regain rate to prevent binge eating or self-induced vomiting during the weight regain process after the competition.

The average age at which athletes began WL practices in the present study was 14.4 years, younger than that reported in most previous studies (3). National-level athletes began WL significantly later than local-level athletes, a pattern that aligns with previous findings in boxing (37). Studies have shown that intentional energy deficits and dehydration during training and competition in childhood or adolescence can disrupt metabolic and hormonal regulation affecting growth, maturation, body composition, menstrual cycles, and reproductive capacity (11, 54, 65, 66). Another study found that female judo athletes who experienced WL during the development of secondary sexual characteristics were significantly shorter in height than those who did not (67). Therefore, it can be hypothesized that an earlier age of WL initiation is associated with potential risks to health and development, which may influence long-term performance outcomes. Practitioners should advise athletes to commence WL at a relatively mature age (such as 17–18 years old) to minimize long-term impacts on growth and sporting career, and to balance the key competitions during adolescence (e.g., those at 17–18 years old, which determine eligibility for adult competitions and university entry).

Findings from this study indicate that coaches were typically the primary figures guiding athletes in WL practices (64%), followed by self-guidance (62%). This aligns with previous research showing that most coaches directly guided the WL processes of their athletes (4, 7, 61, 68). Notably, only ten athletes reported receiving guidance from nutritionist, and only four from doctor during their usual WL process. Considering the high magnitude of WL in this study (>5%), relying solely on coaches or self-guidance may pose potential risks (69, 70). A recent study found that Chinese CS coaches primarily based their WL guidance on their own experiences as athletes (61). Insufficient nutrition knowledge is common among both coaches and athletes (71, 72), and this factor exacerbates the health risks associated with WL (73, 74). For example, in the present study, 49% of athletes perceived that WL had no impact on health, while 21% considered it beneficial. Coaches and athletes may focus primarily on BM itself, without fully understanding differences in body composition or the physiological consequences of different WL methods and rates. As a result, they may be unable to select strategies that are both safe and capable of maintaining or enhancing performance while minimizing health risks. However, their primary reasons for WL were competing against lighter opponents and optimizing athletic performance. These goals place high demands on the selection of WL methods, the transition between methods, the alignment of WL strategies with training loads, and the balance of WL magnitude and nutritional intake. Failure to manage these factors appropriately may undermine the intended benefits of WL. Therefore, strengthening collaboration with nutritionists, doctors, and strength and conditioning coaches is essential, particularly in adolescent and elite sport contexts. For adolescent athletes, ensuring sufficient safety and health are necessary to support proper physical and psychological development, whereas elite athletes require precise performance regulation to achieve marginal gains and secure medals in high-level competitions.

In the present study, despite differences in athletes’ perceptions of WL, their WL practices (e.g., highest WL%, habitual WL%, and WR/WL ratio) did not differ significantly. Similar observations have been reported in previous studies (7). Furthermore, another study examining athletes’ nutrition knowledge and WL behaviors found no significant association between the two (75), suggesting that athletes’ perceptions of WL may not be a key determinant, and that they tend to engage in similar WL practices regardless. This phenomenon may be related to the pervasive culture and experiential transmission of WL in CS. Specifically, Chinese CS athletes are often influenced by coaches, peers, and established training and competition routines, and this cultural and experiential transmission may lead athletes to adopt similar WL strategies even when their individual perceptions differ (4, 36, 37). It should be noted that the WL practices (e.g., highest WL% and habitual WL%) observed in this and previous studies on Chinese athletes differ from those reported in most other countries (3), which may further indicate the potential role of cultural factors. However, due to the lack of data on WL perceptions from athletes in other countries, no statistical inference can be made, and this cultural explanation should therefore be regarded as a descriptive and hypothetical interpretation.

In this study, 18% of athletes presented with ED symptoms, a prevalence higher than Chinese female adolescent CS athletes (6%) surveyed in off-season (7), and similar or lower than that reported in previous surveys of female adolescent athletes from other sports using EDE-Q (18–35%) (76–81). The relatively low prevalence may be due to the off-season timing of the assessment. Given the differing sensitivities of tools used to measure ED, this study compared only results from research employing the EDE-Q (53). Unlike previous findings in female adolescent CS athletes (7), the present study identified significant associations between habitual WL practices and ED scores in male adolescent CS athletes. This result aligns with previous research showing that WL variables in CS were related only to post-competition dietary restraint score in males seven days after the competition, with no significant correlations found in females for any DE score (53). Furthermore, DE scores in female athletes have been shown to improve during three weeks following competition (53). These findings suggest that WL is more strongly and persistently associated with ED/DE symptoms in male combat athletes, and this association is observable even in the off-season. Athletes who habitually allocated 15–21 days for WL had significantly higher global ED score than those allocating 4–5 or 8–10 days, indicating that longer periods of WL are associated with higher restraint score. Frequent exposure to prolonged low energy availability and dietary control coincides with longer WL durations in the present sample. Habitual WL% was also positively associated with eating concern score, which may be due to stricter control over food intake among athletes with higher habitual WL%. Moreover, athletes with higher habitual WL% tended to allocate longer durations for WL, so that in most of the sample, both factors are associated with higher restraint score, eating concern score, and global ED score. These findings hold key practical implications for practitioners: they should prioritize short-term, moderate WL strategies (e.g., limiting WL duration to 4–10 days and reducing habitual WL%) and implement year-round ED screening for male athletes, given the persistent link between prolonged, high-magnitude WL practices and elevated ED symptoms even in the off-season. Considering the significant association between habitual WL%, allocated WL duration, and ED symptoms, and the similarity of habitual WL% and allocated WL duration in the present study to those reported in Chinese female adolescent CS athletes (7), these variables represent the most important basis for comparison. Other factors, such as training environment, culture, and geographic context, were also comparable between the two studies. Taken together, these similarities further support the existence of sex differences in the effects of WL practices on ED symptoms. Future research should further investigate the mechanisms underlying gender differences in the effects of habitual WL practices on ED/DE symptoms.

This study has some limitations. First, the cross-sectional design represents a key constraint, as it only captures a snapshot of the relationships between WL practices, perceptions, and ED symptoms at a single time point. This design prevents establishing of temporal sequences between variables, and thus cannot confirm the direction of associations—for example, whether prolonged WL practices precede elevated ED scores, or whether pre-existing ED symptoms drive restrictive WL behaviors. Longitudinal studies are therefore needed to clarify the dynamic and potentially bidirectional relationships among these variables over time. Second, it relied on retrospective self-reported data, which may have introduced recall and social desirability biases. Future studies should consider more objective approaches, such as using calibrated weight scales to record daily or pre- and post-training BM changes. Third, a critical limitation is that the EDE-Q8 was exclusively administered to athletes reporting engagement in WL, while athletes with no WL experience were excluded from this assessment. This exclusion severely limits the interpretation of ED symptom prevalence in the sample: the reported 18% prevalence of ED symptoms cannot be generalized to the broader population of male adolescent combat sports athletes, nor can meaningful comparisons be made between athletes with and without WL experience regarding the prevalence and severity of ED symptoms. Fourth, this study adopted a convenience sampling method. Although the sample size was large, there might still be some limitations in terms of sample representativeness in certain aspects, such as geographical location and sport discipline composition.

## 5 Conclusion

The magnitude of WL among Chinese male adolescent CS athletes was higher than that reported in previous studies. Most athletes engaged in long-term WL, typically allocating more than 15 days, primarily through increased exercise and the use of plastic suits. During the WL process, coaches were the primary source of guidance, whereas few athletes reported receiving guidance from doctors or nutritionists. The primary reason for athletes engaging in WL was to compete against lighter opponents to increase their likelihood of winning. Higher ED scores were associated with greater habitual WL% and longer allocated WL durations.

## Data Availability

The raw data supporting the conclusions of this article will be made available by the authors, without undue reservation.
