# Supplementary material for: Weight loss practices, perceptions, and eating disorder symptoms among Chinese male adolescent combat sports athletes
Source: Front Nutr. 2026 Jan 22;12:1726260. doi: 10.3389/fnut.2025.1726260 (PMC12875282; doi:10.3389/fnut.2025.1726260)
Supplement: Supplementary file 1 [file Table_1.docx]

**Appendix A**

Table A1. Statistical analysis methods used in the present study to assess the differences or correlation between weight loss practice, perception, and eating disorders variables.

| Dependent variable | Data type | Independent variables | Data type | Method |
| --- | --- | --- | --- | --- |
| Age began WL practices | Continuous | Competitive level | Categorial | Kruskal-Wallis’ tests |
|  |  | Sports discipline | Categorial | Kruskal-Wallis’ tests |
|  |  | Whether the coach guided WL progress | Categorial | Mann-Whitney U test |
| Highest WL% | Continuous | Competitive level | Categorial | Kruskal-Wallis’ tests |
|  |  | Sports discipline | Categorial | Kruskal-Wallis’ tests |
|  |  | Perception of the impact of WL on health | Categorial | Kruskal-Wallis’ tests |
|  |  | Perception of the impact of WL on performance | Categorial | Kruskal-Wallis’ tests |
|  |  | Perception of the impact of fairness on performance | Categorial | Kruskal-Wallis’ tests |
|  |  | Whether the coach guided WL progress | Categorial | Mann-Whitney U test |
|  |  | Age | Continuous | Spearman’s rank correlation test |
|  |  | Age began WL practices | Continuous | Spearman’s rank correlation test |
|  |  | WL experience | Continuous | Spearman’s rank correlation test |
|  |  | Number of WL last year | Continuous | Spearman’s rank correlation test |
| Habitual WL% | Continuous | Competitive level | Categorial | Kruskal-Wallis’ tests |
|  |  | Sports discipline | Categorial | Kruskal-Wallis’ tests |
|  |  | Perception of the impact of WL on health | Categorial | Kruskal-Wallis’ tests |
|  |  | Perception of the impact of WL on performance | Categorial | Kruskal-Wallis’ tests |
|  |  | Perception of the impact of fairness on performance | Categorial | Kruskal-Wallis’ tests |
|  |  | Allocated WL time | Categorial | Kruskal-Wallis’ tests |
|  |  | Whether the coach guided WL progress | Categorial | Mann-Whitney U test |
|  |  | Age | Continuous | Spearman’s rank correlation test |
|  |  | Age began WL practices | Continuous | Spearman’s rank correlation test |
|  |  | WL experience | Continuous | Spearman’s rank correlation test |
|  |  | Number of WL last year | Continuous | Spearman’s rank correlation test |
| Number of WL last year | Continuous | Competitive level | Categorial | Kruskal-Wallis’ tests |
|  |  | Sports discipline | Categorial | Kruskal-Wallis’ tests |
|  |  | Whether the coach guided WL progress | Categorial | Mann-Whitney U test |
|  |  | Age | Continuous | Spearman’s rank correlation test |
|  |  | WL experience | Continuous | Spearman’s rank correlation test |
|  |  | Number of competitions participated last year | Continuous | Spearman’s rank correlation test |
| WR% after competition | Continuous | Habitual WL% | Continuous | Spearman’s rank correlation test |
|  |  | WL experience | Continuous | Spearman’s rank correlation test |
| WR/WL ratio | Continuous | Competitive level | Categorial | Kruskal-Wallis’ tests |
|  |  | Sports discipline | Categorial | Kruskal-Wallis’ tests |
|  |  | Perception of the impact of WL on health | Categorial | Kruskal-Wallis’ tests |
|  |  | Perception of the impact of WL on performance | Categorial | Kruskal-Wallis’ tests |
|  |  | Perception of the impact of fairness on performance | Categorial | Kruskal-Wallis’ tests |
|  |  | Whether the coach guided WL progress | Categorial | Mann-Whitney U test |
|  |  | Age | Continuous | Spearman’s rank correlation test |
|  |  | WL experience | Continuous | Spearman’s rank correlation test |
|  |  | Habitual WL% | Continuous | Spearman’s rank correlation test |
| Perception of the impact of WL on health | Categorial | Sports discipline | Categorial | Chi-square |
|  |  | Competitive level | Categorial | Chi-square |
| Perception of the impact of WL on performance | Categorial | Sports discipline | Categorial | Chi-square |
|  |  | Competitive level | Categorial | Chi-square |
| Perception of the impact of fairness on performance | Categorial | Sports discipline | Categorial | Chi-square |
|  |  | Competitive level | Categorial | Chi-square |
| restraint score | Continuous | Competitive level | Categorial | Kruskal-Wallis’ tests |
|  |  | Sports discipline | Categorial | Kruskal-Wallis’ tests |
|  |  | Allocated WL time | Categorial | Kruskal-Wallis’ tests |
|  |  | WL experience | Continuous | Spearman’s rank correlation test |
|  |  | Habitual WL% | Continuous | Spearman’s rank correlation test |
|  |  | Habitual WL% during 1day before weigh-in | Continuous | Spearman’s rank correlation test |
| eating concern score | Continuous | Competitive level | Categorial | Kruskal-Wallis’ tests |
|  |  | Sports discipline | Categorial | Kruskal-Wallis’ tests |
|  |  | Allocated WL time | Categorial | Kruskal-Wallis’ tests |
|  |  | WL experience | Continuous | Spearman’s rank correlation test |
|  |  | Habitual WL% | Continuous | Spearman’s rank correlation test |
|  |  | Habitual WL% during 1day before weigh-in | Continuous | Spearman’s rank correlation test |
| shape concern score | Continuous | Competitive level | Categorial | Kruskal-Wallis’ tests |
|  |  | Sports discipline | Categorial | Kruskal-Wallis’ tests |
|  |  | Allocated WL time | Categorial | Kruskal-Wallis’ tests |
|  |  | WL experience | Continuous | Spearman’s rank correlation test |
|  |  | Habitual WL% | Continuous | Spearman’s rank correlation test |
|  |  | Habitual WL% during 1day before weigh-in | Continuous | Spearman’s rank correlation test |
| weight concern score | Continuous | Competitive level | Categorial | Kruskal-Wallis’ tests |
|  |  | Sports discipline | Categorial | Kruskal-Wallis’ tests |
|  |  | Allocated WL time | Categorial | Kruskal-Wallis’ tests |
|  |  | WL experience | Continuous | Spearman’s rank correlation test |
|  |  | Habitual WL% | Continuous | Spearman’s rank correlation test |
|  |  | Habitual WL% during 1day before weigh-in | Continuous | Spearman’s rank correlation test |
| Global ED score | Continuous | Competitive level | Categorial | Kruskal-Wallis’ tests |
|  |  | Sports discipline | Categorial | Kruskal-Wallis’ tests |
|  |  | Allocated WL time | Categorial | Kruskal-Wallis’ tests |
|  |  | WL experience | Continuous | Spearman’s rank correlation test |
|  |  | Habitual WL% | Continuous | Spearman’s rank correlation test |
|  |  | Habitual WL% during 1day before weigh-in | Continuous | Spearman’s rank correlation test |
| ED symptom | Categorial | WL experience | Continuous | Mann-Whitney U test |
|  |  | Habitual WL% | Continuous | Mann-Whitney U test |
|  |  | Habitual WL% during 1day before weigh-in | Continuous | Mann-Whitney U test |

Note: WL, weight loss; WR, weight regain; ED, eating disorder; EDE, eating disorder examination;
